# ‘I felt I belonged’: A qualitative study of role modelling and team integration as key drivers of primary care career choice

**DOI:** 10.1080/13814788.2025.2527143

**Published:** 2025-07-11

**Authors:** Eva Pfarrwaller, Camille Laurent, Johanna Sommer, Anne Baroffio, Dagmar M. Haller, Hubert Maisonneuve

**Affiliations:** a Faculty of Medicine, University Institute for Primary Care, University of Geneva, Geneva, Switzerland; b Faculty of Medicine, University College of General Medicine, University Claude Bernard Lyon 1, Lyon, France; c Unit of Development and Research in Medical Education, Faculty of Medicine, University of Geneva, Geneva, Switzerland

**Keywords:** Primary care, undergraduate medical education, clinical placements, career choice, role modelling

## Abstract

**Background:**

Clinical placements significantly impact medical students’ career choices. Primary care physicians supervising these placements can influence students’ career decisions through role modelling and by creating supportive learning environments.

**Objectives:**

This qualitative study aimed to identify factors contributing to role modelling and students’ sense of integration during placements and their influence on career decisions, with a focus on primary care.

**Methods:**

Semi-structured interviews were conducted with postgraduate trainees selected based on interest in primary care, exploring their experiences during undergraduate clinical placements and factors influencing career choices. Data were thematically analysed to identify key themes related to student integration, well-being, and supervisor role modelling.

**Results:**

Analysis revealed four key domains where primary care physicians can positively influence students’ career interest: onboarding students effectively, fostering positive and inclusive team dynamics, involving students in patient care, and providing high-quality supervision and feedback. Students reported that feeling valued and socially included contributed to their well-being and professional self-efficacy, which in turn impacted their career choices.

**Conclusion:**

Supportive and inclusive learning environments during placements are critical to fostering students’ professional growth. While relevant across clinical settings, this study’s findings hold particular significance for primary care due to the challenge of balancing clinical and practice management duties and teaching. Implementing structured onboarding, team integration, and effective supervision can enhance students’ experiences and promote interest in primary care. Future research should extend these findings beyond primary care. The proposed roadmap could both spark interest in primary care and promote future collaboration between primary and secondary care.

## Introduction

High-functioning primary care is widely recognised as the foundation for effective health care systems, particularly given the increasing prevalence of chronic conditions [1,2]. A large and well-trained primary care workforce is essential to achieve the five aims for healthcare improvement: better population health, enhanced patient experience, cost control, workforce well-being, and health equity [3]. In undergraduate medical education, robust evidence supports the role of primary care placements in increasing the visibility and attractiveness of primary care as a career option, an key step towards building a high-performing workforce that meets population health needs [4–6]. Clinical placements help students align their personal interests and needs with various career options [7]. Research shows that placements have the greatest impact on career choice when they are of high quality [4,8].

It is crucial to identify the right quality indicators in these placements: A common pitfall is to focus on easily measurable factors, such as the acquisition of content knowledge. However, recent studies suggest that informal and emotional factors, including student well-being, play a significant role in shaping positive placement experiences and influencing career decisions [9]. One such factor is role modelling, which affects students’ career choices in a more subtle, informal way [10]. Role models are defined as ‘individuals admired for their ways of being and acting as professionals’ [11], and research has shown a link between identifying a positive role model and students’ well-being during placements [12]. Primary care physicians (PCPs) may not always realise the extent of their influence on students’ career decisions through role modelling, especially in ways that go beyond explicit professional behaviours associated with clinical and teaching excellence.

We previously developed a conceptual framework based on the concepts of professional interests and individual decision-making processes to study medical students’ primary care career choices [13]. This framework aims to support efforts to enhance the attractiveness of primary care as a career. We then conducted a qualitative study to test the framework’s applicability in real-world settings [14]. That study highlighted the central role of clinical learning in shaping students’ career choices through personal experiences and exposure to different work environments, and it suggested that placements significantly influence the affective component of students’ professional self-efficacy, particularly their sense of being integrated into a team.

The present study seeks to deepen our understanding of how students’ sense of integration and role modelling interact to shape their placement experiences. We hypothesise that students’ well-being and sense of integration within the practice team are linked to their perception of the supervising physician as a role model, and that this interaction influences their career choices.

To address this, we used a qualitative approach guided by the following research questions:

- What factors contribute to students’ sense of integration during a placement?
- How do these factors relate to positive or negative role modelling by the supervising clinician?
- How do these factors influence career decision-making, and are they specific to primary care?
- What actionable recommendations can be drawn for the primary care teaching community?

## Methods

### Approach and design

We interviewed young physicians in early postgraduate training to retrospectively explore their experiences during undergraduate medical education, collecting career choice narratives linking past experiences to their current professional roles [15]. The study’s data collection and analysis were grounded in a constructivist paradigm and guided by a previously developed conceptual framework [13]. This framework conceptualises students’ career choices as an ongoing interaction between cognitive elements, such as self-efficacy, and decision-making within a hierarchical system of external influences.

### Participants

We employed a two-step sampling strategy, gathering data from two distinct contexts (Switzerland and France, details in Table 1). First, we recruited physicians in their first postgraduate training year who had graduated from the Faculty of Medicine at the University of Geneva, Switzerland, in 2018. This cohort was tracked throughout their undergraduate medical education as part of a study on academic performance and career choice [16,17]. Based on these cohort data, and aligned with our goal of identifying factors related to primary care career choice, we used purposive sampling to select participants who had expressed an interest in primary care during their studies. Of the 33 potential participants invited, 14 were interviewed [14].

**Table 1.** Main features of study participants’ educational and primary care contexts.

	Switzerland (University of Geneva)	France (various universities)
Undergraduate medical education	Six-year curriculum: Year 1: Pre-selection year (mostly basic sciences) Years 2 and 3: Pre-clinical curriculum. Early clinical experience in primary care (4 half-days). Years 4 and 5: Clinical rotations (mandatory placements in different specialties, mostly hospital-based). Placement in primary care practice (8 half-days). Year 6: Elective year (10 months, of which 1 month mandatory in primary care). Final exam without national ranking	Six-year curriculum (curriculum determined on a national level): Year 1: Pre-selection year (mostly basic sciences) Years 2 and 3: Pre-clinical curriculum. Early clinical experience in primary care depending on medical schools Years 4, 5, and 6: Clinical rotations (mandatory placements in different specialties, mostly hospital-based). Mandatory placement in primary care practice (3 to 8 weeks). National Ranking Exam at the end of year 6 and subsequent residency matching process
Postgraduate training	Unrestricted choice of any specialty after graduation Overseen by physicians’ professional organisation Training mostly organised by trainees themselves by applying to training positions offered by hospitals (a few formal training programs available) Duration minimum 5 years, mostly hospital based. Six months mandatory training in outpatient settings (possibility of training opportunities in primary care practices)	Restricted choice of specialty depending on residency matching process Under the responsibility of medical faculties, respecting a national reference framework drawn up by the ministries of higher education and health Training organised by medical faculties, in accordance with regional health authorities Duration 3 years, minimum 12 months in primary care
Primary care training	Primary care physicians have postgraduate training in mostly hospital-based, general internal medicine (‘family medicine track’) or paediatrics. At the end of their training, they practice in an outpatient/private practice setting.	Primary care physicians have postgraduate training in general practice and practice in an outpatient/private practice setting.

Secondly, we expanded our sample by recruiting young physicians in their first to third postgraduate training years from various locations in France (Angers, Bordeaux, Lyon, Marseille, Mayotte, Montpellier, and Tours). This sample, comprising individuals already enrolled in primary care postgraduate training, was recruited using snowball sampling. One author (CL) contacted 10 acquaintances; six responded and recommended seven potential participants not personally known to CL, all of whom agreed to participate.

### Data collection

We collected career choice narratives and experiences related to clinical placements through semi-structured interviews. Swiss participants were interviewed in person by EP between May 2019 and January 2020, while French participants were interviewed via video conference by CL between January and April 2021. The interview guide, informed by our conceptual framework, began with participants’ reflections on how they reached their current professional roles and then explored career-related decisions and personal experiences. For the French participants, the guide was adapted to emphasise experiences during clinical placements, particularly their sense of team integration and the influence of role modelling (see Supplementary Appendix). All interviews were conducted in French, audio-recorded, transcribed verbatim, and anonymised.

### Analysis

Transcripts were coded using Atlas.ti software [18], following a mixed inductive-deductive approach guided by the Framework method [19]. The initial interviews were coded inductively to generate a list of codes, which was then iteratively applied to subsequent interviews. After each batch of three or four interviews, the research team reviewed and refined the emerging themes. Codes and related citations were then analysed to understand the relationships between themes. Data collection continued until thematic saturation was achieved, meaning that no new codes or themes emerged. Swiss participants’ interviews were coded by EP and CL, while French participants’ interviews were coded by CL, along with two research assistants (a French junior physician and a Swiss medical student).

### Researcher characteristics and reflexivity

EP led the Swiss part of the study and has a background in paediatrics and public health, with a research focus on primary care career choice. The French part of the study was conducted by CL as part of her MD thesis in France [20], under the supervision of HM, who is a PCP and academic based in both France and Switzerland. The analysis and synthesis of results were discussed extensively within the research team, which also included primary care professors experienced in research and clinical supervision (DMH and JS) and a professor of medical education research and development (AB). This research was also informed by well-established frameworks of primary care career choice [13] and role modelling [12], supporting the external validity of the study.

The reporting of the study adheres to the Standards for Reporting Qualitative Research (SRQR) [21].

### Ethical considerations

The Swiss portion of the study was exempt from formal ethical review by the Ethical Review Board of the Canton of Geneva, in accordance with Swiss laws on research involving human subjects. For the French part of the study, formal ethical approval was not required as the study did not fall under the legal definition of research involving human participants. All participants were informed about the audio recording, transcription, anonymisation, and data analysis processes and provided written consent before the interviews.

## Results

**Figure 1.**
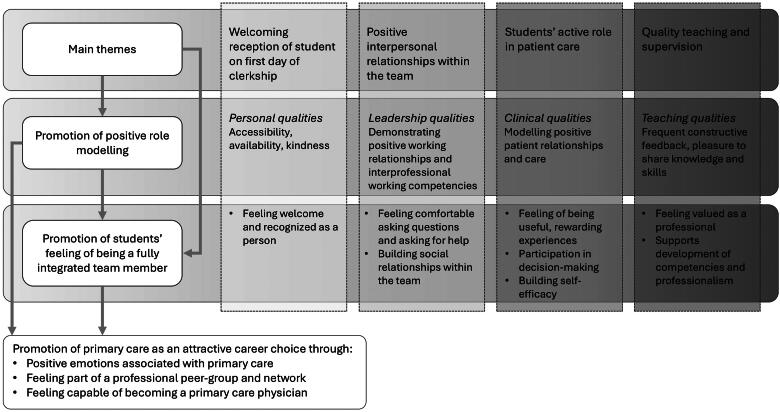
Illustration of the four main themes identified in the analysis and how actions within these themes may increase the likelihood of students choosing a career in primary care through positive role modelling and their sense of belonging to the team. The grey shading illustrates the physician’s stepwise engagement related to the actions suggested in Table 3.

Table 2 presents participants’ characteristics. The interviews lasted between 34 and 56 min (mean 46 min). The analysis of their narratives revealed four major themes that influenced their sense of team integration during placements: (1) Onboarding students through a welcoming reception, (2) experiencing positive team relationships, (3) having an active role in patient care, and (4) benefitting from high-quality teaching and supervision. Within each theme, key elements and actions emerged through which physicians promoted positive role modelling and student integration, ultimately contributing to students’ professional self-efficacy and career projections (Figure 1).

**Table 2.** Participants’ characteristics.

Participant no.^a^	Age (years)^b^	Gender	Country	Postgraduate training year (PGY)^b^ Projected career choice
01	26	M	Switzerland	PGY 1 Gynaecology/Obstetrics or dermatology
02	26	F	Switzerland	PGY 1 General internal medicine (primary care)
03	23	F	Switzerland	PGY 1 Internal medicine subspecialty
04	26	F	Switzerland	PGY 1 Paediatrics (primary care)
05	23	F	Switzerland	PGY 1 Gynaecology/Obstetrics
06	28	M	Switzerland	PGY 1 General internal medicine (primary care)
07	27	F	Switzerland	PGY 1 General internal medicine (primary care)
08	26	F	Switzerland	PGY 1 Paediatric subspecialty
09	26	F	Switzerland	PGY 1 Hospital internal medicine
10	27	F	Switzerland	PGY 1 Oncology
11	25	M	Switzerland	PGY 1 General internal medicine (primary care)
12	25	M	Switzerland	PGY 1 Paediatrics (primary care)
13	25	F	Switzerland	PGY 1 Paediatrics (primary care)
14	25	F	Switzerland	PGY 1 Neurology
15	27	F	France	PGY 3 General practice
16	24	F	France	PGY 1 General practice
17	26	F	France	PGY 3 General practice
18	31	M	France	PGY 3 General practice
19	27	M	France	PGY 2 General practice
20	29	M	France	PGY 3 General practice
21	29	F	France	PGY 3 General practice

a Participants were numbered consecutively according to interview sequence.

b At the time of interview.

1. ***Onboarding students:*** Many participants described the first day of a placement as pivotal, where actions, such as being introduced to the team, becoming familiar with the practice, and being assigned clear roles contributed to their sense of inclusion and well-being. Essential interpersonal gestures, such as supervisors knowing students’ names, further reinforced this feeling.
*“For me, it was important to be seen as an individual, not just as the student. Having clear tasks and a designated point of contact was crucial. The worst placements were those where every other day we had a different supervisor.” (P7)*
*“We had a welcome day where we were introduced to the whole team—secretaries, doctors, residents, nurses, nurse assistants, everyone. The physician in charge welcomed us, she asked us to call her by her first name, and gave us her phone number so we could call her if there was any issue. It made everyone feel comfortable.” (P16)*
  In contrast, negative experiences, often reported from hospital placements, highlighted a lack of structured introductions and defined tasks, leaving students disoriented.
*“I really didn’t have a role to play at all. It was even less than just observation because many times I didn’t know who to follow. I struggled to understand what my role was supposed to be, what I was actually supposed to do.” (P3)*
2. ***Positive team relationships:*** Participants frequently cited team dynamics as an important influence. Teams characterised by respect, kindness, and mutual aid fostered a safe learning environment in which students felt comfortable asking questions. Positive role models were often noted for their leadership qualities and ability to cultivate a respectful team atmosphere. Social bonds, such as shared meals or informal interactions, also contributed to a strong sense of belonging.
*“Respect is key. Not all supervisors treat students with respect. It’s also about having a good atmosphere outside of work; we were invited to share meals with the team. Also, it’s about not being judged for not knowing something. Even if, as students, we didn’t know all the answers, I never felt judged for it.” (P15)*
3. ***Active role in patient care:*** An active role beyond mere observation was crucial for learning and feeling integrated. Supervisors who involved students in patient interactions, decision-making, and clinical tasks were seen as positive role models. Such involvement made students feel useful and connected to the work environment and clinical practice.
*“He tried to create a triangle, not just a straight line with me as an observer. At the end of the patient interview, he’d ask me, ‘Do you have any questions? Is there something you’d like to clarify with the patient?’ Sometimes, the patient would even turn to me and address me directly instead of the doctor. It made me feel truly involved.” (P2)*
4. ***Teaching and supervision:*** High-quality feedback was central to positive experiences. Frequent, constructive feedback helped students improve their skills and fostered a non-judgmental environment where they felt valued. A strong feedback culture contributed to effective role modelling, with supervisors remaining approachable and available for guidance. Participants recalled teams with such a culture favourably due to the emphasis on the learning process.
*“It was mostly about theoretical knowledge. The supervisors asked me questions, guided me through diagnostic reasoning and the differential diagnosis. They supervised me during clinical exams, giving tips on how to improve, and eventually let me conduct the exams on my own. Afterward, we’d go over the findings together. It was a very hands-on approach.” (P14)*
*“The supervisor took the time to teach us, explaining how to properly admit a patient, build a problem list, and approach each issue. If we didn’t fully understand something, like heart failure that we had only studied in theory, he took time to explain it practically, sometimes even drawing diagrams. He was always available to answer questions, and there was no judgment. We felt comfortable asking questions. His availability, combined with his expertise and willingness to teach, made all the difference.” (P9)*

Figure 1 illustrates the interrelation of the four themes, with role modelling and students’ sense of integration reinforcing each other. Participants’ narratives suggest that this interplay significantly impacted their learning, professional self-efficacy, and career projections. Positive role models in patient care, leadership, and teaching instilled confidence, enabling students to envision future careers in the specialties they observed.

*“My general practice rotation went really well, and one of the doctors I worked with completely reignited my passion for medicine. She was a young doctor, a mother of two kids, always dynamic, cheerful, and kind. It was a revelation for me—she practiced exactly how I had imagined in my mind.” (P21)*

Feeling valued, receiving constructive feedback, and taking on meaningful roles helped students develop professional self-efficacy. This confidence encouraged autonomy in clinical work and made students feel capable.

*“[Regarding a placement in primary care] I felt grown up! Yes, I felt good, autonomous, and valued. Because sometimes, during hospital placements, you feel useless, like you don’t know anything and aren’t contributing. But there, I felt like I was useful and that I actually knew things.” (P16)*

*“When I was in primary care paediatrics, the doctor gave me his chair and said, ‘Alright, this patient is coming in for this reason. How are you going to structure your interview?’ I’d explain my plan, and he’d say, ‘That’s good, but you could try this differently.’ It made me realize that things weren’t as inaccessible as I’d thought. In other placements, we weren’t always allowed to step into the doctor’s role, to gain confidence in ourselves as physicians. Some rotations left me feeling like, ‘I don’t want to do this because I don’t feel capable,’ but that was because I wasn’t given the chance to build confidence. It really depends on the supervisor.” (P8)*

Participants often linked these positive experiences to their career choices, envisioning themselves in specialties where they had strong role models. Conversely, negative team dynamics or lack of integration led some students to reconsider previously favoured specialties.

*“It was a summer placement [in primary care] that lasted about a month, and it was great. I still remember the doctor I worked with; she taught me so much, especially about patient interviews and how to conduct a consultation, which I had never done before. I was really focused on my studies, so it was interesting to exchange knowledge. We had some complicated cases and had to seek second opinions, which gave me insight into the connection between general practice and hospital medicine, between generalists and specialists. We got along really well, and it made me realize I was genuinely interested in general practice. It opened my mind to the possibility of choosing that specialty.” (P20)*

Enthusiastic and passionate physicians emerged as particularly strong role models, motivating students by demonstrating fulfillment in their specialty.

*“He [an infectious diseases specialist] really left an impression on me, especially for his professionalism and his teaching. He made complex topics, like antibiotics, seem completely logical and easy to grasp, even though the textbook on infectious diseases felt overwhelming. He had an incredible sense of humour and often shared stories from his work. He was fascinating from start to finish.” (P18)*

## Discussion

The core finding of this study is that physicians who supervise students play a crucial role in shaping their career choices by acting as positive role models and enhancing students’ overall well-being during placements. While these dynamics are evident in all clinical settings, they may be particularly pronounced in primary care due to its unique structure and environment. Physicians influence students not only through individual actions but also through their overall attitude and deliberate integration of students into the practice, thereby creating a positive learning atmosphere. Based on our findings, we identified four key areas in which PCPs can actively foster interest in primary care: welcoming or onboarding students, promoting integration and team relationships, involving students in patient care, and providing quality supervision and teaching. In Table 3, we present a roadmap of actionable elements derived from the four themes that emerged from our study. This roadmap outlines a progression from easily implementable, high-impact actions to formal training for more complex supervisory skills.

**Table 3.** Suggested action plan for successfully integrating students into primary care practices.

Stepwise action plan based on main themes		Suggested actions to implement	Implementation tips
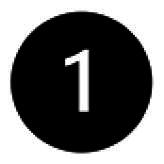	Welcoming reception of student on first day of placement (onboarding)	Schedule a dedicated time (at least 30 min) for student onboarding Create a welcoming environment Set the stage for the placement	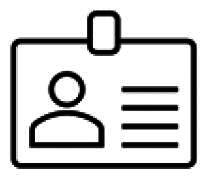 Memorise key student information (name, year of study)
			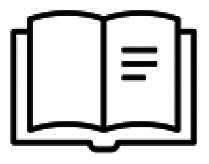 Prepare a summary welcome document announcing the student’s arrival to team and patients
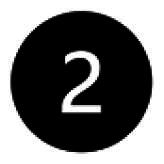	Positive interpersonal relationships and integration within the team	Formally introduce students to the team and clarify their role Model positive team communication and collaboration	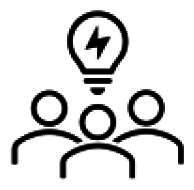 Develop leadership and care coordination skills
			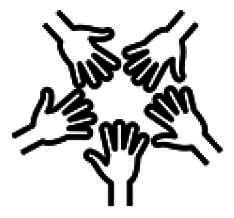 Apply evidence-based teamwork tools
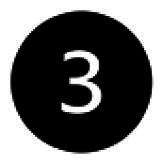	Students’ active role in patient care	Create frequent opportunities for students to actively engage in patient care Identify tasks that students can carry out and define appropriate level of supervision	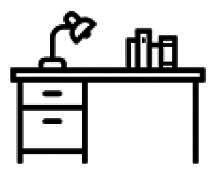 Arrange a separate consultation room for student, if possible
			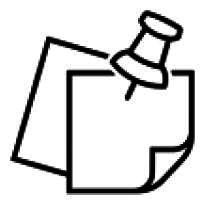 Alternatively, arrange administrative tasks to work on while student consults
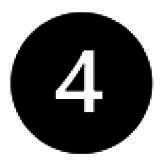	Quality teaching and supervision	Provide frequent feedback on students’ performance Start with direct supervision and evolve to indirect supervision as appropriate	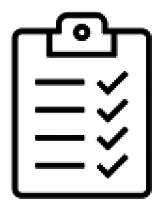 Rely on tools to support quality of supervision (e.g. 1 min preceptorship, think aloud, priming)
			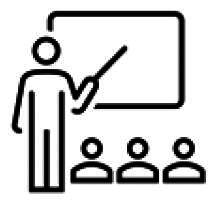 Formal training in clinical supervisory skills

Our findings extend existing literature by demonstrating that student well-being and sense of belonging significantly influence career choices [14]. A positive placement environment supports constructive learning, as students feel valued both personally and professionally, reinforcing a safe, supportive setting. Similarly, a recent UK study on nursing and allied health students found that enhancing students’ experiences during primary care placements improved team communication and collaboration [22]. This suggests that investing in student integration not only benefits learning but also strengthens team functioning.

When students are given active roles, with responsibilities tailored to their skill level and accompanied by constructive feedback, they build the confidence they need to progress. This active engagement, combined with quality supervision, allows them to practise what they observe in role models [12] and contributes to their professional self-efficacy, a key factor in career decision-making [13,23].

Our findings are consistent with a recent realist literature review from the United Kingdom exploring students’ perspectives on primary care careers [9]. That study concluded that a welcoming and supportive placement environment not only encourages students to view primary care more favourably but also fosters a sense of value that can lead to an emotional attachment to the specialty. These positive experiences prompt career-related reflections, helping students assess how well a specialty aligns with their personal and professional priorities. This further emphasises that placements with supportive mentorship are pivotal not just for primary care but for shaping broader career decisions across various medical specialties.

These reflections on career alignment are closely tied to students’ observations of their supervising physicians. Positive placement experiences not only help students feel valued but also reinforce the role modelling observed throughout their clinical rotations. Our study, along with previous research, confirms that placements are critical for positive role modelling [12,24]. Observing physicians at work and being integrated into healthcare teams enhances students’ sense of value and professional self-efficacy [25]. While existing frameworks categorise role modelling into personal, clinical, and teaching domains [26], our findings suggest that these domains are often interconnected in practice, influenced by physicians’ leadership and teaching qualities. Role modelling occurs implicitly and continuously through patient consultations, supervision, and team interactions. Conversely, a negative team environment can hinder the recognition of even the most skilled physicians as role models. Ideally, supervisors should provide trainees with a safe space for experiential learning, both physically and emotionally, to foster trust and inclusion [27].

Our participants described experiences from both hospital and primary care placements. While their experiences in primary care were predominantly positive, potentially suggesting that PCPs excel at engaging students, our interviews indicate that key activities (such as active involvement in patient care, quality supervision, and a welcoming attitude) occurred in both settings. This finding suggests that larger student groups in hospitals may limit opportunities for personalised teaching and role modelling, highlighting the importance of organisational factors like student-to-teacher ratios. Our stepwise action plan (Table 3) is designed to be adaptable across settings, with a specific focus on the unique opportunities in primary care.

### Strengths and limitations

One strength of our study is the two-step sampling strategy, which allowed us to first explore career choice processes broadly and then focus on specific factors. Including participants from both Switzerland and France enhanced the transferability of our findings. We acknowledge that relying on participants’ recollections may introduce memory bias. While a prospective approach would provide a more comprehensive, longitudinal view, our method enabled participants to link past experiences with their current roles and thus construct their own narratives.

Our study population was chosen to better understand how to support primary care career choices in undergraduate medical education, in line with our mission to train and strengthen the future primary care workforce. We recognise that our findings are not entirely specific to primary care and may apply to clinical placements in other specialties; however, further studies with students uninterested in primary care are needed to confirm this.

### Implications

Previous research has highlighted the need for guidance to help PCPs integrate students effectively during placements [22,25], while also suggesting that they may have limited time for formal faculty development [28]. The framework in Table 3 offers a practical reference for PCPs aiming to enhance student experiences during placements. The proposed actions reflect key professional values, such as empathetic and supportive relationships, clear interprofessional communication, and accessibility, and contribute to team well-being, a prerequisite for healthcare improvement [3,29]. Investing in positive role modelling and student experiences during placements is likely to influence overall team well-being and career choices, ultimately enhancing health system performance.

Although our study focused on participants who were already inclined towards primary care, our recommended practices are accessible to a broad range of learners. The key activities suggested in our roadmap (Table 3) not only help foster an early interest in primary care but also promote future collaboration at the primary care–secondary care interface. Our conceptual framework [13] and related research [30,31] suggest that cultivating the interest in primary care is a dynamic process that involves constants, such as longitudinal exposure, key ingredients like the four elements identified in our study, and adaptive elements that address students’ evolving priorities, from early altruistic considerations to more personal and professional drives at a later stage in medical education.

## Conclusion

Primary care physicians have a unique opportunity to create supportive, enriching learning environments that enhance students’ well-being and professional self-efficacy, ultimately shaping their ability to envision a career in primary care. Although integrating students into a busy practice can be challenging, our study suggests that engaging them in patient care and serving as positive role models can bridge the gap between clinical work and teaching. We propose practical, stepwise actions contributing to the training of future primary care professionals and fitting within the busy schedules of PCPs. The roadmap supports student engagement and career interest while also establishing principles that foster collaboration across the primary–secondary care interface.

## Supplementary Material

Supplemental Material

## Prior presentations

The study’s outcomes were presented as a conference abstract at the AMEE 2024 Conference in Basel, Switzerland.
