# Supplementary material for: ‘I felt I belonged’: A qualitative study of role modelling and team integration as key drivers of primary care career choice
Source: Eur J Gen Pract. 2025 Jul 11;31(1):2527143. doi: 10.1080/13814788.2025.2527143 (PMC12258163; doi:10.1080/13814788.2025.2527143)
Supplement: Supplemental Material [file IGEN_A_2527143_SM6368.docx]

Appendix to the manuscript titled **“I felt I belonged”: A qualitative study of role modelling and team integration as key drivers of primary care career choice**

Interview Guide used for the French part of the qualitative study. The original guide in French was translated to English using a generative AI tool (ChatGPT 4.0, OpenAI) and then reviewed and edited by the first author.

**Introduction and information phase**

- Thank the person for their participation. Presentation: Junior physician, end of internship in late 2020 (highlighting proximity to the participant). Ask if an informal address is acceptable.
- Icebreaker question = How is the internship going, for example, adapted to the current semester.
- Brief information about the study: determinants of career choice in medicine, with a focus on certain influences (do not go into detail to avoid influencing responses).
- Information about the interview: recording, anonymized data, allowed to not answer certain questions, possibility to stop interview at any time; take your time to think before answering a question, especially if it calls for older memories.
- Verify that the consent form has been signed.
- Ask if there are any questions about how the interview will proceed.

**Main Interview Phase**

Start the recording (inform participant).

| ***What I need to know*** | ***Questions to ask*** | ***Clarifications and follow-ups*** |
| --- | --- | --- |
| Internship specialty and career projection | To start, you are a general medicine intern, in which semester are you currently and what rotation are you doing?  And how do you envision your practice, say in 10-15 years? (In a hospital? In outpatient care? In the city or countryside?) |  |
| Desired specialty before the ranking examination influenced the choice | Did you have another specialty in mind when making your final choice? Maybe before incorporating the ranking results, which can sometimes be decisive for some people? |  |
| Career choice timeline: specialty and type of practice (before, at the start, and at the end of medical school) | Do you remember when you decided you wanted to pursue this specialty?  Before starting your studies, did you already have an idea?  You told me you envision yourself in (hospital or outpatient care). Do you remember when you made this choice? Is it something you always knew, or is it more recent? | *If the choice was made late:*  Did you have another specialty in mind at the beginning or during your studies before making this choice? |
| Memorable placements | Do you remember any placements during your studies that particularly stood out to you? It could be either positive or negative.  Do you remember other placements? | *If yes but doesn’t elaborate:*  Could you tell me more about this placement? How did it go? What did you like or dislike?  *If no:*  Do you remember placements you did in your current specialty? In specialties you hesitated with? In specialties you excluded from your choice? |
| Role during placements | What was your role as a trainee during these placements?  Did you participate in patient care? In what way? | *If no active role or administrative role:*  How did you feel about this role?  *If active role:*  How did you know what you were supposed to do?  How did you feel in this role during this placement?  How did it feel to have this role with responsibilities, having a place in patient care?  How did the doctors intervene in your work? |
| Reception and relationship with team members | Could you tell me more about your first days of the placement? How were you welcomed?  What did you feel after this reception?  How did things go with the team members during the placement? | What does a good reception/bad reception mean to you concretely?  What made you have a good/bad relationship with them? How did they behave? |
| Exposure to role models | During your placements, do you remember a particular person or several people who made an impression on you? Either positively or negatively?  Someone you took as an example or counterexample? | *If yes:*  Could you describe these people, what you liked or disliked about them?  How did they act with you? With their colleagues? With patients?  How did they organize their work time?  *Depending on the specialty:* Did this encounter with this person create doubts about your specialty choice? |
| Disappointments during placements / negative experiences | Do you remember placements that disappointed you or during which you had a hard time? | *If yes:*  Could you tell me more? What did you dislike?  Was it the work itself? The type of diseases? Or more the atmosphere of the placement or the people? |
| Influences of placements on career choice | Based on what you’ve mentioned, are there placements that confirmed your choice of a specialty or ruled one out?  Are there placements that, on the contrary, changed your view of a specialty?  For example, considering a specialty in your choice when it wasn’t part of it before?  Or finally excluding a specialty that you had considered until then? |  |
